# CD1d expression on chronic lymphocytic leukemia B cells affects disease progression and induces T cell skewing in CD8 positive and CD4CD8 double negative T cells

**DOI:** 10.18632/oncotarget.10372

**Published:** 2016-07-01

**Authors:** Nadja Zaborsky, Franz Josef Gassner, Daniela Asslaber, Petra Reinthaler, Ursula Denk, Sabine Flenady, Josefina Piñón Hofbauer, Barbara Danner, Stefan Rebhandl, Andrea Harrer, Roland Geisberger, Richard Greil, Alexander Egle

**Affiliations:** ^1^ Laboratory for Immunological and Molecular Cancer Research, Department of Internal Medicine III with Haematology, Medical Oncology, Haemostaseology, Infectiology and Rheumatology, Oncologic Center, Paracelsus Medical University, Salzburg, Austria; ^2^ Salzburg Cancer Research Institute, Salzburg, Austria; ^3^ Department of Neurology, Paracelsus Medical University, Salzburg, Austria

**Keywords:** CLL, T cells, T cell skewing, CD1d, CD161

## Abstract

Chronic lymphocytic leukemia develops within a complex network driven by genetic mutations and microenvironmental interactions. Among the latter a complex interplay with the immune system is established by the clone. Next to a proposed recruitment of support from T and myeloid cells, potential anti-CLL immune reactions need to be subverted.

By using TCL1 mice as a CLL model, we show that TCR-Vβ7^+^ NK1.1^+^ T cells are overrepresented in this disease model and constitute a main subset of peripheral CD3^+^ cells with biased TCR usage, showing that these cells account for a major part for T cell skewing in TCL1 mice. Moreover, we show that overrepresentation is dependent on CD1d expression in TCL1 mice, implicating that these cells belong to a NKT-like cell fraction which are restricted to antigen presented by the MHC-like surface marker CD1d. Accordingly, we observed a high fraction of CD161^+^ cells within overrepresented T cells in CLL patients and we found downregulation of CD1d on the surface of CLL cells, both in TCL1 mice and patients. Finally, we show that in TCL1 mice, CD1d deficiency resulted in shortened overall survival. Our results point to an interaction between CLL and CD161^+^ T cells that may represent a novel therapeutic target for immune modulation.

## INTRODUCTION

While immunotherapy has been under evaluation for many decades, only recent advances in effectively modulating the patient's immune system have made immunotherapy a promising and appealing strategy in cancer treatment. With its intricate dysregulation of the T cell system, chronic lymphocytic leukemia (CLL) may be an especially interesting target disease for immunotherapeutic approaches [1]. While the use of immune modulatory drugs such as lenalidomide, and the use of therapeutic antibodies, which target inhibitory immune receptors, were recently shown to partly overcome CLL-associated immune defects in patients and CLL mouse models [2–7], their mode of action, as well as the exact subsets of immune cells targeted by these compounds, still remains elusive.

In this study, we asked whether CD1d restricted T cells are involved in immune perturbations and T cell skewing associated with CLL and whether these cells would thus qualify as target for immune reconstitution during therapy. CD1d restricted T cells comprise a T cell subset which is activated by lipid antigen presented by the monomorphic MHC class I like surface molecule CD1d. Generally, these cells are grouped as natural killer (NK) T cells which constitute a subset of innate T cells which share features of both innate and adaptive immunity [8–9]. NKT cells can respond very rapidly to activation with the production of various cytokines (IFNγ, IL-4, IL-10, IL-13, IL-17, IL-21) and tumor necrosis factor, thereby enhancing or suppressing immune responses in an MHC independent manner [10]. Unlike MHC class I/II, CD1d presents glycolipid antigens such as α-Galactosylceramide (α-GalCer), which can be derived from self or non-self antigenic determinants. Generally, NKT cells are defined by the expression of the NK cell marker NK1.1 (mouse) or CD161 (human) on CD3^+^ T cells and are divided into type I and type II NKT cells [11]. While type I NKT cells harbor a conserved invariant TCR (thus giving rise to the term invariant or iNKT), preferably consisting of TCR-Vβ11 in combination with an invariant Vα24 in humans and TCR-Vβ2, TCR-Vβ7, TCR-Vβ8.2 in mice, type II NKT cells express a more diverse TCR repertoire [12]. In CLL, CD1d has recently been shown to be aberrantly expressed and its expression level was predictive for a poor clinical outcome [13–14]. Concomitantly, CD56^+^ T cells, which are an NKT-like population, were shown to be decreased in progressive CLL patients [15]. In parallel, many T cell dysfunctions were described to occur in CLL and the occurrence of mono- and oligoclonal T cell expansions have been observed alongside CLL development in human patients as well as in the TCL1 mouse model for this disease [16–20].

In this study, we aim at a better definition of skewed T cells in CLL. While our previous results show that in human CLL skewed and overrepresented CD4^+^ T cells consist of PD-1^+^ exhausted T cells [19], our data presented in this manuscript suggest that overrepresented CD8^+^ as well as CD4/CD8 double negative (DN) T cells frequently belong to the CD161^+^ CD3^+^ T cell compartment. Similar to human CLL, leukemic TCL1 mice show severe TCR-Vβ skewing of NK1.1^+^ CD3^+^ T cells, which is abrogated when TCL1 mice are bred on a CD1d deficient background. Finally, CD1d deficiency resulted in reduced overall survival of leukemic mice.

## RESULTS

### TCR-Vβ usage is skewed in T cells from TCL1 mice

**Figure 1:**
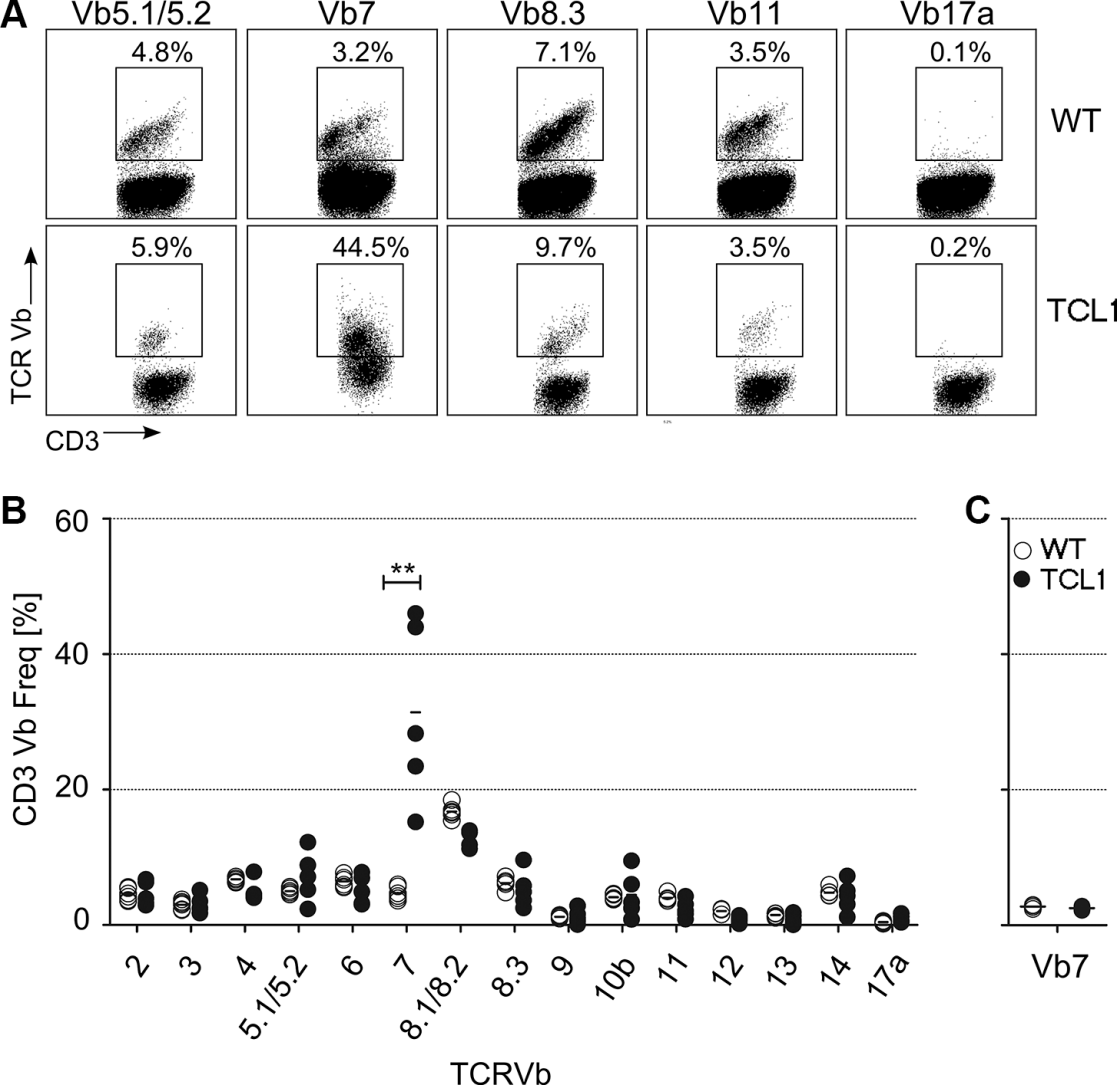
TCR-Vβ usage in the TCL1 CLL mouse model Splenocytes from sacrificed leukemic TCL1 mice and from age-matched wildtype (WT) mice were stained using CD3 and TCR-Vβ-specific antibodies. (**A**) Representative FACS plots for WT and TCL1 mice are shown. (**B**) Graph showing percentage of CD3^+^ T cells from leukemic mice, which are expressing the respective TCR-Vβ element (WT *n* = 6; TCL1 *n* = 5). (**C**) Graph showing percentage of CD3^+^ T cells from young preleukemic mice (age ≤ 150 days), which are expressing the TCR-Vβ7 element (*n* = 4). (Horizontal bars indicate mean percentage).

**Figure 2:**
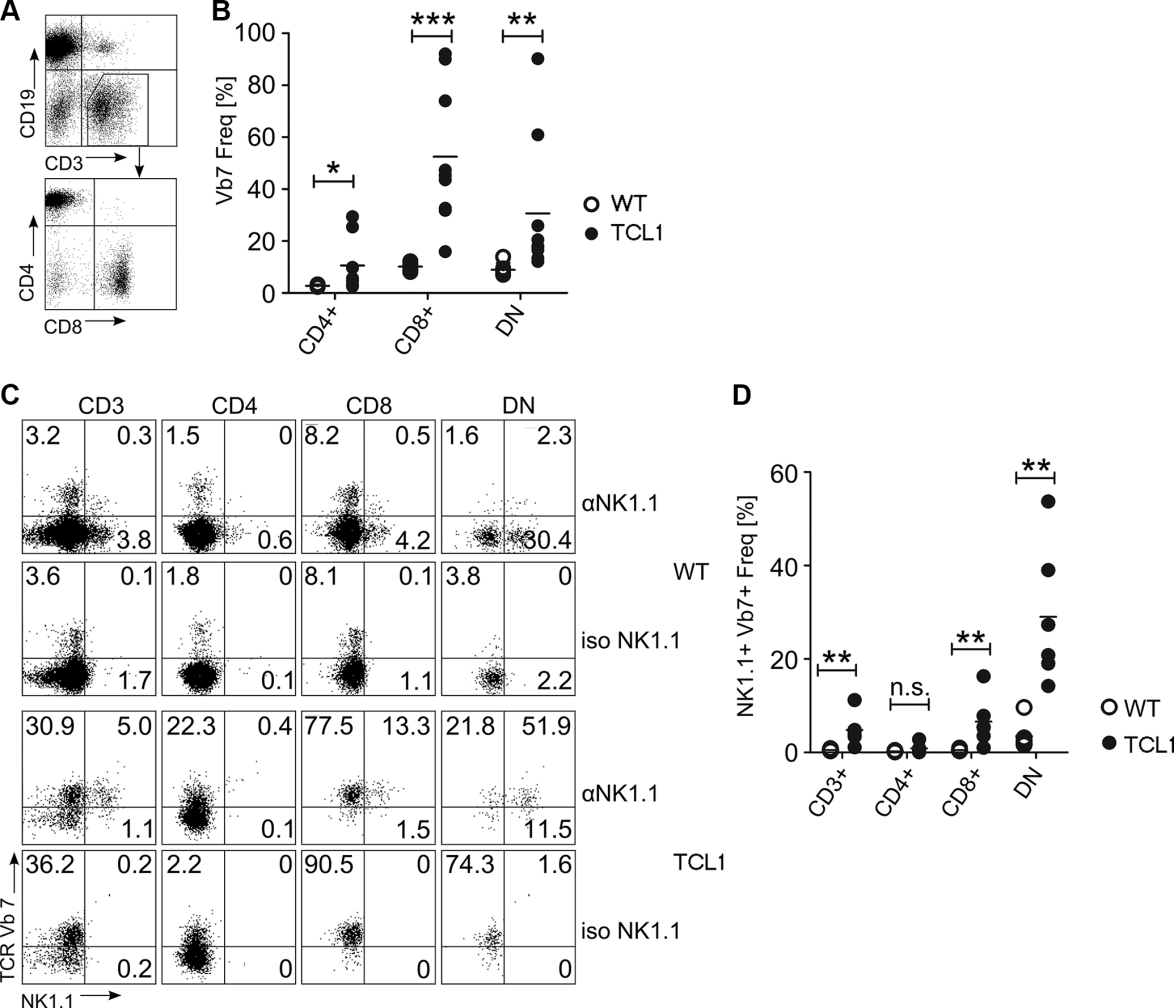
TCR-Vβ7 usage in T cell subsets of the TCL1 mouse CD3^+^Vβ7^+^ T cells from TCL1 mice were further stained for CD4 and CD8 expression (**A, B**) and for NK1.1 (**C, D**). Representative FACS profiles and graphs showing statistical analysis are shown. WT: *n* = 6 (B and D), TCL1: *n* = 9 (B) or *n* = 6 (D). (DN: double negative for CD4 and CD8; iso: staining using an isotype control antibody instead of an anti-NK1.1 antibody). (Horizontal bars indicate mean percentage).

We determined TCR-Vβ usage in splenocytes from five fully leukemic TCL1 animals and compared the results to six age-matched wild type mice. We found that CD3^+^ T cells expressing the TCR-Vβ7 element were dramatically overrepresented in leukemic animals (Figure 1A, 1B; 4.7% ± 0.95% vs 31.4% ± 13.3%; *p* = 0.004; Mann-Whitney test), while no apparent difference was observed for other Vβ-specific CD3^+^ T cells (Figure 1A, 1B). Notably, Vβ7 overrepresentation was dependent on leukemia development, as young preleukemic animals did not show enrichment of TCR-Vβ7 T cells (Figure 1C). By staining the Vβ7^+^CD3^+^ T cells of sacrificed leukemic mice with antibodies for CD4 and CD8, we further found that these T cells were specifically enriched within CD8^+^ and CD4/CD8 double negative (DN) T cell fractions (Figure 2A, 2B; for CD4^+^ T cells: 2.8% ± 0.3% vs 10.6% ± 9.9%; *p* = 0.016; for CD8^+^ T cells: 10.2% ± 1.7% vs 52.5% ± 26.8%; *p* = 0.0004; for DN cells: 8.9% ± 2.6% vs 30.6% ± 26.8%, *p* = 0.0016; Mann-Whitney test). As Vβ7 is a TCR-Vβ chain commonly used by NKT cells in mice [21], we additionally stained these cells for expression of NK1.1, a marker typically expressed by NK and NKT cells. In comparison to wild type animals, we found that leukemic animals showed a high fraction of the CD8^+^ and DN Vβ7^+^ T cells that was positive for NK1.1 (Figure 2C, 2D; CD3^+^Vβ7^+^ cells: 0.5% ± 0.2% vs 4.8% ± 3.4% *p* = 0.005; CD3^+^CD4^+^Vβ7^+^ cells: 0.2% ± 0.2% vs 0.9% ± 1.0% *p* = 0.084; CD3^+^CD8^+^Vβ7^+^ cells: 0.5% ± 0.2% vs 6.6% ± 5.3% *p* = 0.005; CD3^+^DN Vβ7^+^: 3.5% ± 3.1% vs 29.0% ± 14.8% *p* = 0.002; Mann-Whitney test).

### CD161 cells are enriched in CLL patients

**Figure 3:**
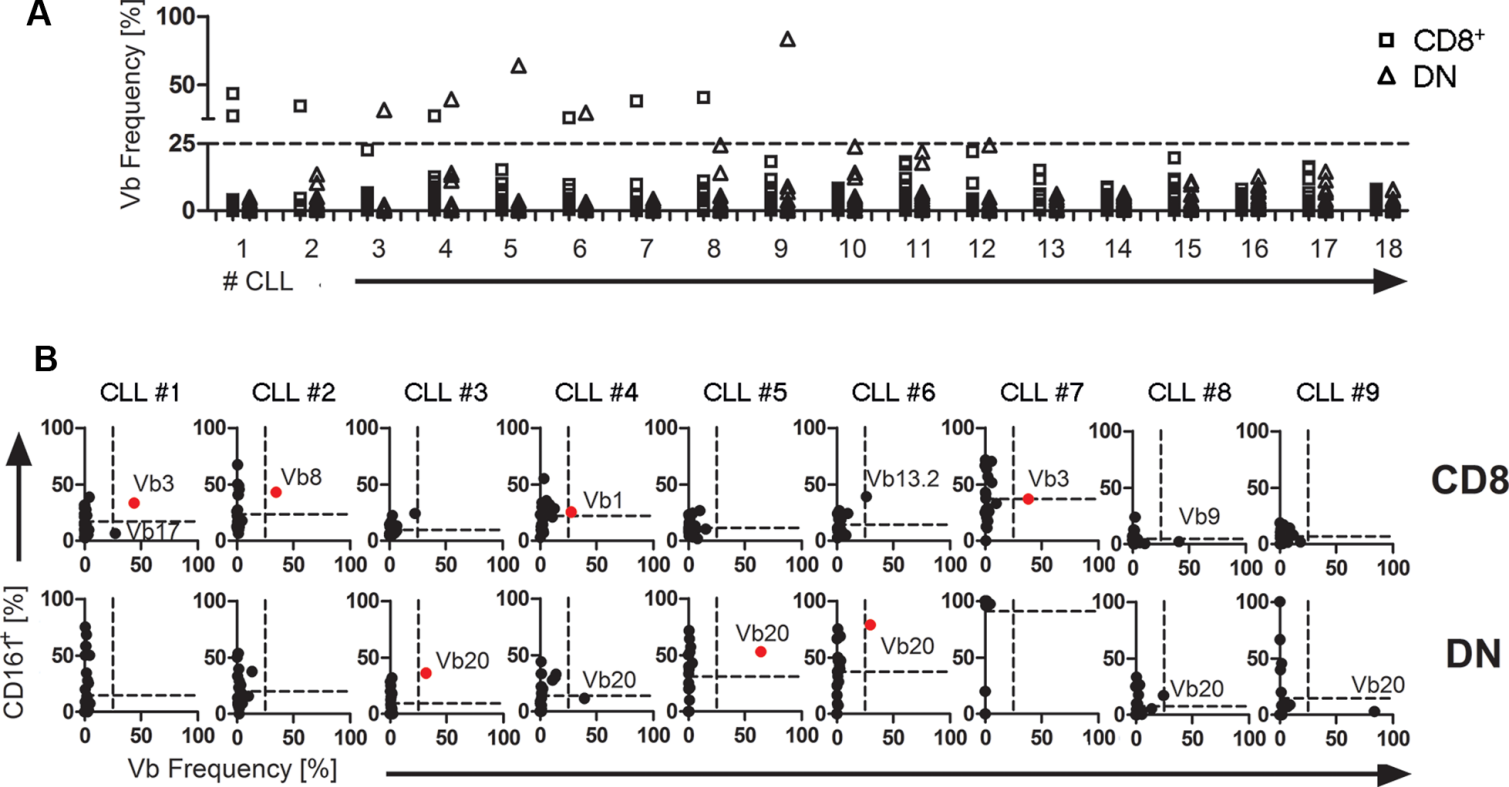
CD161 expression and TCR-Vβ skewing in human CLL TCR-Vβ usage and CD161 expression within CD8^+^ or DN T cells (CD3^+^) were quantified by flow cytometry from peripheral blood of 18 CLL patients. (**A**) The frequency of 24 distinct TCR-Vβ elements within CD8^+^ (square) or DN (triangle) T cells (CD3^+^) were analysed for 18 CLL patients. A ticked horizontal line at 25% marks the threshold for overrepresentation. (**B**) For nine CLL samples with overrepresented T cells, expression values for 24 TCR-Vβ elements were plotted against CD161 expression. A TCR-Vβ usage of more than 25% was considered overrepresented (the 25% threshold is indicated by a vertical ticked line within each plot). The mean percentage of CD161^+^ cells of all TCR-Vβ subsets is shown as horizontal ticked lines. TCR-Vβ-specific T cells within the upper right quadrant (shown in red) are defined as overrepresented T cells with substantial CD161 expression. (the respective overrepresented Vβ element is indicated within each plot).

We next investigated whether in line with our results from TCL1 mice, CLL patients exhibit an increased percentage of CD161^+^ cells within overrepresented T cell clones. We therefore stained peripheral blood lymphocytes from 18 consecutive non-selected CLL patients using CD161 and TCR-Vβ-specific antibodies. In line with our previous results [19], we found that in the peripheral blood of some CLL patients, overrepresented TCR-Vβ-specific T cells could be discerned, reaching up to > 80% occurrence within the peripheral T cell pool (Figure 3A). Using an arbitrary cut-off of ≥ 25% occurence of T cells using a particular Vβ element, we found that from 18 consecutive CLL samples analysed, 9 showed at least one overrepresented CD8^+^ or DN Vβ-specific T cell fraction. In 7 out of these 9 cases with overrepresented T cells, at least one of the respective T cells exhibited a substantial expression of CD161 which was above the mean CD161 expression levels of all TCR-Vβ-specific T cells (CLL #1–#7; Figure 3, Supplementary Table S1). Among the remaining two samples, one had a dominant DN TCR-Vβ20 fraction at borderline frequency of 24,5% with clear CD161 expression (CLL #8, Figure 3) and only one CLL sample showed a dominant T cell clone without CD161 expression (CLL #9, Figure 3). Strikingly, within the DN T cell fraction, all overrepresented cells expressed the TCR-Vβ20 element (Figure 3B).

Of note, in contrast to TCL1 mice, we could not detect a general increase of CD161^+^ or CD56^+^ T cells in CLL patients compared to healthy volunteers (not shown) and the overrepresented T cells were not restricted to expression of a particular Vβ element but showed a diverse set of Vβ elements with a bias towards Vβ20 usage in DN T cells (Figure 3). Notably, in our small cohort analysed, we did not observe an apparent correlation of clinical parameters including CMV seropositivity with the presence or absence of overrepresented CD161^+^ T cells (Supplementary Table S1).

### CD1d expression is altered on CLL samples from patients and from TCL1 mice

**Figure 4:**
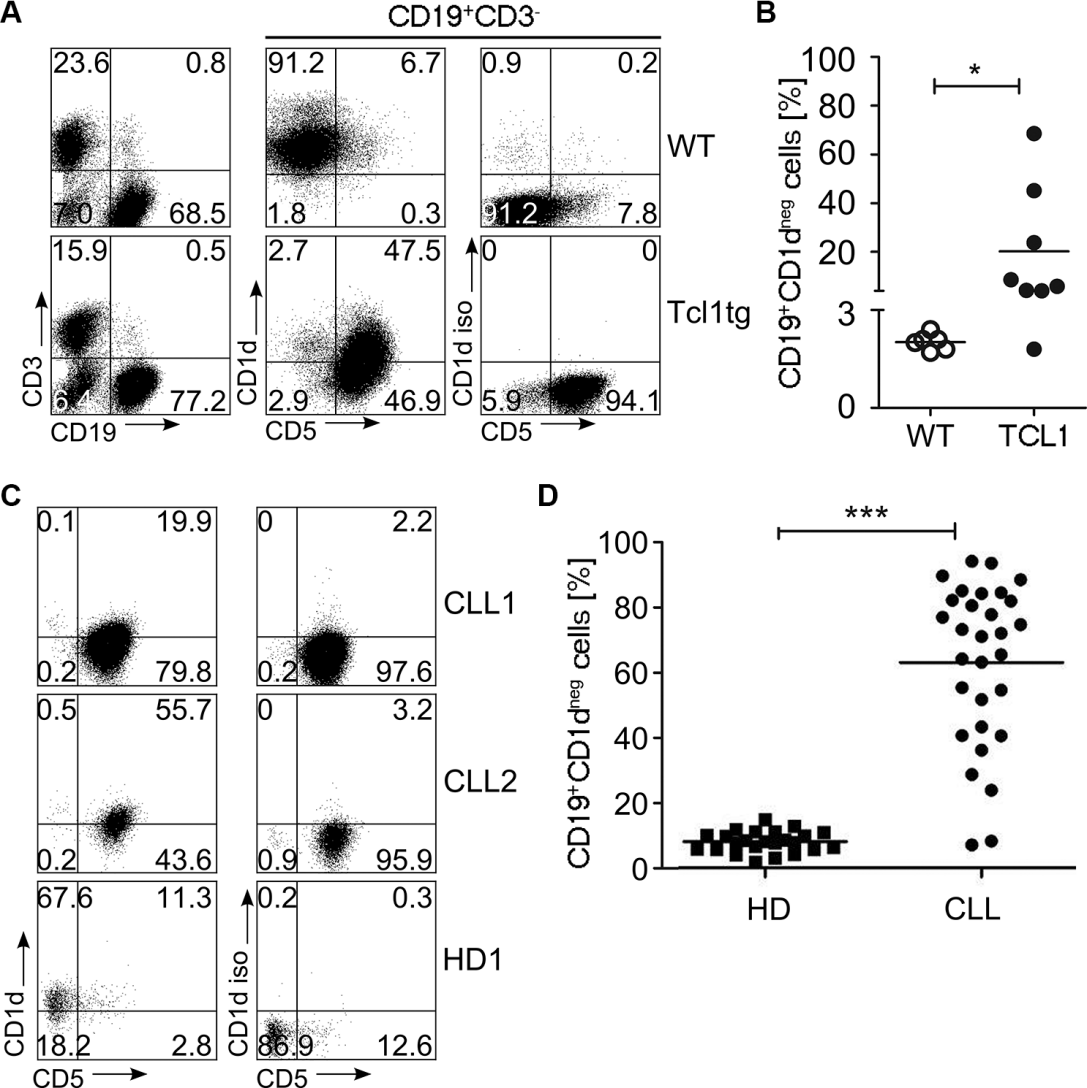
CD1d expression in TCL1 mice and human CLL samples CD1d was measured on the surface of TCL1 and wildtype (WT) mouse splenocytes. (**A**) A representative FACS plot is shown. (**B**) Shown is the fraction of CD1d negative samples among CD19^+^ WT B cells (*n* = 6) and the tumor cell population of TCL1 mice (*n* = 8). (**C**) A representative FACS stain of CD1d on the surface of human CLL samples and B cells from healthy controls (HD) is shown. (**D**) The fraction of CD1d negative cells for CLL (*n* = 30) and HD (*n* = 24) was determined as shown in (A). (Horizontal bars indicate mean percentage).

As at least a fraction of CD161^+^ T cells belong to the NKT cell compartment which is restricted to the MHC-like surface protein CD1d, we next determined whether CD1d expression is altered alongside NK1.1^+^ T cell augmentation in TCL1 mice. We therefore stained CLL cells from leukemic mice for CD19, CD5 and CD1d and determined CD1d expression levels compared to B cells from wildtype mice by flow cytometry. As shown in Figure 4, we found that leukemic B cells had significantly downregulated surface CD1d as defined by the percentage of cells within the gate for cells stained with an isotype control antibody (CD1d negative CLL cells; Figure 4). Hence, while in wild type animals almost all B cells expressed CD1d (percentage of CD1d negative cells: 2.0% ± 0.2%), leukemic cells from TCL1 mice uniformely shifted to a significantly lower CD1d expression (percentage of CD1d negative cells: 20.3% ± 24.4%; Mann Whitney test *p* = 0.014) (Figure 4A, 4B). To further correlate the frequency of NKT cells with the downregulation of CD1d on CLL cells in the TCL1 mouse model over time, we stained blood samples from several mice at pre-leukemic and leukemic stage and monitored NKT cell levels and CD1d expression. We observed a significant correlation of CD1d negative leukemic cells with the occurrence of Vb7^+^NK1.1^+^ DN T cells (R^2^ = 0.35 *p* = 0.008; Supplementary Figure S1). To test whether these results were recapitulated in human CLL, we stained consecutive non-selected human CLL samples and B cells from healthy controls for expression of CD1d (patient characteristics are given in Supplementary Table S2). In human CLL samples, we observed an even more dramatic downregulation of CD1d, reflected in a significantly higher CD1d negative cell fraction as well as decreased MFI for CD1d in patients compared to healthy volunteers (Figure 4D and Supplementary S2, CD1d negative cells: 8.0% ± 3.2% vs 63.2% ± 24.8%, Mann Whitney test *p* < 0.0001). Notably, the appearance of CD1d negative CLL cells significantly correlated with advanced RAI stage and with VLA4 subunit CD49d low expression in our patient cohort (Supplementary Table S3). No association was found for CD1d expression and other clinical parameters including CMV status (Supplementary Tables S2 and S3).

To further functionally characterize CD161/NK1.1 positive CD8^+^ and DN T cells from patients and TCL1 mice, we determined the production of IFNγ, IL-4 and TNFα of these cells in presence of leukemic cells loaded with or without αGalCer as CD1d restricted lipid antigen. In TCL1 mice, CD8^+^ T cells expressing these cytokines were specifically enriched within the NK1.1^+^ subset (Supplementary Figure S3A) while in CLL patient samples, DN and CD8^+^ T cells exhibited increased cytokine production within the CD161^+^ fraction, except for IL-4 (Supplementary Figure S3B). In both cases, cytokine production was rather independent from incubation with αGalCer as lipid antigen.

### T cell skewing in leukemic mice is dependent on CD1d expression

**Figure 5:**
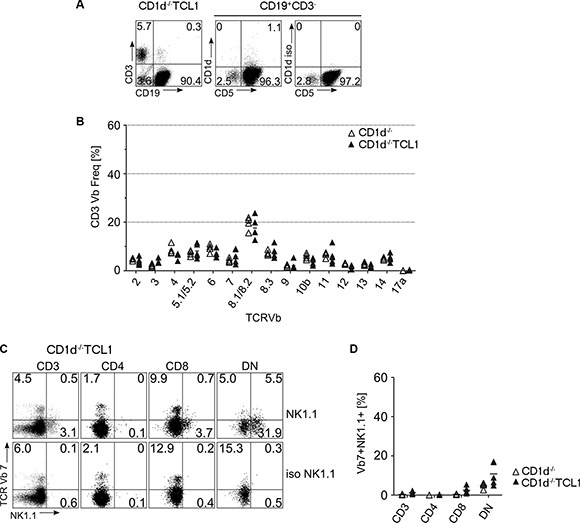
T cell skewing in CD1d knockout mice (**A**) CD1d^−/−^ TCL1 mice generate CLL similar to CD1d^+/+^TCL1 mice. Depicted is a representative FACS stain from a CD1d^−/−^ TCL1 mouse on splenocytes stained for CD3, CD19, CD5 and CD1d. On the middle and right FACS plot, CD5 and CD1d expression of gated CD3^−^CD19^+^ cells are depicted. (**B**) TCR-Vβ usage was determined in CD1d^−/−^ (*n* = 4) and CD1d^−/−^ TCL1 (*n* = 5) mice as described for Figure 1. (**C, D**) CD3^+^Vβ7^+^ T cells from CD1d^−/−^ TCL1 mice were further stained for CD4 and CD8 expression and for NK1.1. Representative FACS profiles (C) and graphs (D) are shown. (DN: double negative for CD4 and CD8; iso: staining using an isotype control antibody instead of an anti-NK1.1 antibody). (Horizontal bars indicate mean percentage).

To test whether CLL development as well as concomitant skewing of CD8 and DN T cells was dependent on CD1d, we bred TCL1 mice onto an CD1d-deficient background. We observed that CD5^+^CD19^+^ CLL cells accumulated in spleens of CD1d^−/−^ TCL1 mice (Figure 5A). However, staining splenocytes of sacrificed leukemic mice with TCR-Vβ-specific antibodies revealed that the severe T cell skewing that is usually associated with CLL development in TCL1 mice was absent in CD1d^−/−^ TCL1 mice, especially within the DN compartment (Figure 5B and Supplementary Table S4). Though a mild skewing was observed in Vβ7^+^NK1.1^+^ CD8^+^ and DN subsets of CD1d^−/−^ TCL1 mice (Figure 5C, 5D), TCR-Vβ7 skewing within the DN subset was significantly different between CD1d deficient and proficient TCL1 mice (*p* = 0.017; Supplementary Table S4).

### CD1d deficiency shortens overall survival of leukemic mice

**Figure 6:**
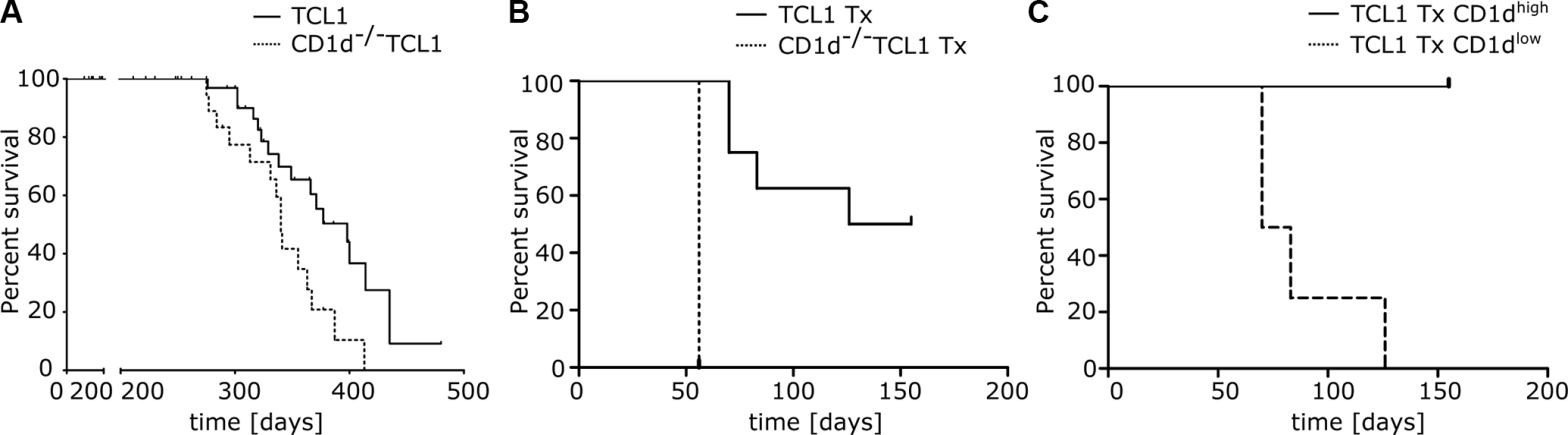
Overall survival in CD1d proficient and deficient TCL1 mice (**A**) CLL development was monitored in CD1d^+/+^TCL1 (*n* = 47) and CD1d^−/−^ TCL1 (*n* = 26) mice and overall survival was assessed for both cohorts. (**B**) Overall survival of wildtype recipient mice transplanted (Tx) with TCL1 and CD1d^−/−^ TCL1 tumors (CD1d^−/−^ TCL1 Tx *n* = 4 and TCL1 Tx *n* = 8). (**C**) Overall survival of wildtype recipient mice transplanted with TCL1 tumors shown in (B; TCL1 Tx). Mice were grouped in two cohorts based on CD1d expression in peripheral blood (TCL1 Tx CD1d^high^ *n* = 4 and TCL1 Tx CD1d^low^ *n* = 4) and percent survival was calculated.

Finally, we tested whether the rate of CLL development is dependent on CD1d expression. We monitored CD1d proficient and deficient TCL1 mouse cohorts for signs of illness and sacrificed mice at standardized humane endpoints. While the median overall survival for CD1d^+/+^TCL1 mice was 398 days (*n* = 47), we observed a significantly shortened overall survival of 340 days for CD1d^−/−^ TCL1 mice (*n* = 26; Log-rank Mantel Cox test *p* = 0.011; Hazard Ratio 0.352; 95% Confidence Interval = 0.157–0.788; Figure 6A). Concomitantly, CD1d^−/−^TCL1 mice showed an accelerated leukemia development in the peripheral blood (Supplementary Figure S4) with no difference in spleen weights at time of sacrifice (Supplementary Figure S5).

To further corroborate that CD1d deficiency accelerates CLL development, we transplanted primary tumors from CD1d proficient and deficient TCL1 mice into wildtype recipient mice. To better assess the influence of CD1d in the transplant setting, we chose a primary TCL1 tumor with high percentage of CD1d expression (Supplementary Figure S6A left panel). We observed that recipient mice transplanted with CD1d^−/−^ TCL1 tumors died earlier from leukemia than mice transplanted with TCL1 tumors (CD1d^−/−^ TCL1 *n* = 4; TCL1 *n* = 8; Log-rank Mantel Cox test *p* = 0.0009; Hazard Ratio 61.87; 95% Confidence Interval = 5.405 to 708.2; Figure 6B). Again, we could show that a substantial fraction of CD1d negative CLL cells (from 18% to 78% of CLL cells when sacrificed) accumulated over time in peripheral blood of recipients of cohort #2 transplanted with TCL1 tumors from spleen #249. Mice which downregulated CD1d on CLL cells (recipients receiving spleen #249) showed a faster tumor growth and died significantly earlier from leukemia than mice which preserved high CD1d expression (recipients receiving spleen #250) (TCL1 Tx CD1d^high^ *n* = 4; TCL1 Tx CD1d^low^ *n* = 4; Log-rank Mantel Cox test *p* = 0.0062; Hazard Ratio 20.95; 95% Confidence Interval = 2.376 to 184.7; Figure 6C and Supplementary S6A, middle and right panel; cohort 2 + 3). Notably, TCR-Vβ7 skewing, which we observed in TCL1 mice was not pronounced in our transplant system, possibly due to the increased velocity of tumor growth upon transplantation (Supplementary Figure S6B).

## DISCUSSION

Microenvironmental interactions are important for CLL cells to survive and proliferate [22]. In particular, immune interactions with cognate T cells are suggested to play a significant role in disease pathogenesis, since it was observed that in CLL, the T cell compartment exhibits a range of dysfunctions together with the frequent occurrence of overrepresented Vβ-specific T cells, which are often oligo- or monoclonal [5, 17, 18, 23–26]. Since these T cell perturbances were also observed in other B cell Non-Hodgkin lymphomas (NHLs), it is supposed that the T cell compartment may fundamentally influence disease progression and development of NHLs even though their pathogenesis is extremely heterogeneous [27].

We recently showed that CD4^+^ T cell skewing in CLL is strongly associated with the BCR mutation status and stereotype, further implicating a likely involvement of antigen in the pathogenesis of CLL [19]. While skewing of CD4^+^ T cells as a putative CLL-supportive compartment could reflect CLL-induced changes in CLL/CD4^+^ T cell immune synapse formation [4, 16, 19, 28], abnormalities in CD8^+^ or DN T cells could mirror the attempts of the immune system to cytolytically attack the malignant cells. Hence, a more thorough investigation of skewed CD8^+^ and DN T cells could help to identify putative cancer specific cytolytic T cells, which might be utilized for novel therapeutic approaches.

In this study, we report that in the TCL1 mouse model, overrepresented CD3^+^ T cells are mainly TCR-Vβ7^+^ NK1.1^+^ and CD8^+^ or DN. While NK1.1/CD161 is expressed on diverse T cell subsets [29–30], our observation that this overrepresentation is absent in CD1d-deficient TCL1 mice is a strong evidence that these T cells belong to a CD1d restricted NKT-like subset. Indeed, absence of CD1d on leukemic cells not only abolished TCR-Vβ7 T cell skewing especially within the DN T cell population in TCL1 mice, but also led to reduced overall survival in these mice, implicating CD1d restricted T cells in CLL defence. Accordingly, TCL1 mice showed downregulation of CD1d on the surface of CLL cells which correlated with the occurrence of TCR-Vβ7 specific T cells, likely reflecting a continous selective pressure for CLL cells to evade immune recognition through CD1d restricted T cells. In line with that, CLL cells from patients also showed downregulation of CD1d, likely reflecting the same immunologic pressure through CD1d restricted T cells. Strikingly, our data suggest overexpression of TCR-Vβ7 T cells in TCL1 mice not only in DN and CD8^+^ T cells, but to a lesser extent also in CD4^+^ T cells (Figure 2B). It is conceivable that these overrepresented CD4^+^ TCR-Vβ7 T cells also belong to an NKT subset and are CD1d restricted, possibly even to the same lipid antigen. Indeed, CD1d restricted NKT cells are considered to mainly belong to CD4^+^ or DN subsets, although the existence of CD8^+^ NKT cell populations was reported [31, 32]. Analogously to our mouse data, expanded CD8^+^ and DN T cells in CLL patients showed a high percentage of CD161 expression. In mouse and human, NK1.1/CD161^+^ DN and CD8^+^ T cells showed robust production of NKT specific cytokines irrespective of stimulation with αGalCer. This could imply that these cells are not αGalCer-restricted but rather specific for another CLL specific glycolipid antigen presented on CD1d. However, as CD161 expression is not restricted to NKT cells but is associated with diverse T cell subsets [30, 33, 34], further studies are certainly necessary to test antigen specificity and functional capacities, which is beyond the scope of this study.

While we found a clear CD1d dependent overrepresentation of Vβ7-specific NK1.1^+^ T cells in TCL1 mice, we did not observe Vβ7 skewing despite of CD1d downregulation on leukemic cells in our tumor-transplant system, although a general T cell skewing towards effector-memory T cells was recently reported upon transplanting TCL1 tumors into wildtype recipients [16]. Probably, the lack of Vβ7 skewing in recipients could be due to the accelerated tumor growth upon transplantation. In other words, clonal expansion of tumor specific NKT cells would be dependent on continous interaction with tumor cells over a long time course, which is only the case in primary TCL1 animals as these mice have approximately a three times longer overall survival. Alternatively, other subsets of the recipient's immune system apart from the NKT cells could take lead in fighting off the tumor, impeding Vβ7 skewing in the transplant system.

In contrast to our TCL1 mice, we could not discern a general increase of CD161^+^ T cells in CLL patients. In addition, a recent report showed that CD56^+^ NKT-like cells are decreased in progressive CLL patients [15]. However, measuring NKT subsets based on expression of surrogate markers such as CD56 or CD161 could well yield opposing results. In addition, though we did not observe increased CD161^+^ T cells in patients, we noticed a striking overrepresentation of TCR-Vβ20 specific DN T cells with substantial CD161 expression in this study. While we could not find reports on this particular T cell subset in the literature, it has been shown that Vβ20 specific T cells have a restricted CDR3 length distribution in children with acute B lymphoblastic leukemia [35]. Future studies will certainly be necessary to more thoroughly define CDR3 sequences as well as cytokine profile and functional capacities of these T subset to precisely elucidate their role in CLL pathogenesis.

Concomitant with the high percentage of CD161 expression on overrepresented T cells, CD1d was markedly downregulated in human CLL samples, which might reflect a strategy for immune evasion from CD1d dependent cytolytic attack. Notably, downregulation of CD1d was also observed in murine CLL. As we suspect these T cells to be CLL-specific, it will be important to evaluate the feasibility of utilizing these cells in clinical strategies to control CLL. According to a previous report suggesting CD3^+^CD56^+^ NKT cells to be used for CLL therapy after *in vitro* augmentation [36], it will be important to evaluate TCR-Vβ-specific CD3^+^CD161^+^ cells for that purpose. In particular, it will be interesting to test cytotoxicity of these cells upon modulation of CD1d on CLL cells and whether restoration of CD1d on CLL *in vivo* could be accomplished by immunomodulatory drugs such as lenalidomide, which was recently shown to reconstitute several immune dysfunctions associated with CLL [1, 3, 5, 6]. Of note, it would be interesting to search for CD161^+^ cells or NKT-like phenotypes within overrepresented T cell subsets in other B cell NHL.

In conclusion, we found NK1.1/CD161^+^ cells in a substantial fraction of TCR-Vβ-specific overrepresented T cells in CLL and can show that loss of CD1d in the mouse model significantly accelerates disease development. Thus the observed downmodulation of CD1d in murine and also in human CLL represents a plausible mechanism for immune escape from a CD1d dependent cytolytic T cell attack. In this scenario modulation of CD1d may be a novel goal for CLL immunotherapy approaches.

## MATERIALS AND METHODS

### CLL samples

Peripheral blood from CLL patients was collected at our medical department upon informed consent in accordance with the Declaration of Helsinki and upon approval by the ethics committee of Salzburg, Austria (Ref. No. 415-E/1287/4–2011 and 415-E/1287/8–2011). Peripheral blood from age-matched healthy controls was collected at the stroke prevention center at the department of neurology upon informed consent. The patients' characteristics are shown in Supplementary Tables S1 and S2. The determination of prognostic markers was performed as previously described [37].

### Mice

Animal experiments were performed under approval from the central Austrian animal ethics committee. The licenses for animal experimentation, BMWF 66.012/0009-II/3b/2012 and 20901-TGV/52/11-2012, are held by Alexander Egle. TCL1 mice on C57BL/6 background [16, 38] or backcrossed to CD1d-deficient mice, which lack type I NKT cells (Jackson Laboratories, Bar Harbor, ME, USA; Stock Number: 008881) were followed for signs of illness and killed by CO_2_ suffocation at humane endpoints. Spleen samples for flow cytometric analysis were collected and subsequently stained using the respective antibodies. Transplanation of murine tumor cells into congenic immune competent wildtype recipient mice was performed as previously described [16]. In brief, splenocytes from TCL1 mice and CD1d^−/−^ TCL1 mice were transferred intraperitoneally into recipients (one tumor into four recipients). Leukemia development, CD1d expression, Vβ7 skewing and overall survival was monitored in those mice where transplanatation of tumor cells was successful (ie recipients in which leukemic cells were detectable by flow cytometry on tail vein blood from at least one single bleeding post transplantation).

### Flow cytometry

For the characterization of lymphocytes from CLL patients and healthy donors, fresh blood samples were stained prior to erythrocyte lysis using FACS lysing solution (Becton Dickinson). The following antibodies were used: CD3 FITC, CD19 Brilliant Violet (both Biolegend), CD5 PE-Cy5 (Beckman Coulter), CD8 Pacific Orange (Invitrogen), CD4 PE-Cy7, CD1d PE, CD161 eFluor 450, CD56 PerCP-eFluor 710, CD3 AlexaFluor 700, TCR Vα24 APC (all eBioscience).

For determination of TCR-Vβ usage, cells were stained with the IOTest^®^ Beta Mark Kit (Beckman Coulter) where eight sets of three TCR-Vβ - specific antibodies (each set comprises a FITC, PE and FITC/PE conjugated antibody) are combined in a single test. Samples were stained together with antibodies for CD3, CD4, CD8, TCR Vα24, CD56 and CD161.

Murine samples (splenocytes of sacrificed mice) were stained using the following antibodies: TCR-Vβ FITC (TCR-Vβ screening panel, BD Pharmingen), CD3 PE, CD1d PE (all BD Pharmingen), CD4 PE-Cy5, CD19 PE-Cy7, CD8 Pacific Blue, CD3 AlexaFluor 700, CD5 PE-Cy5, CD8 PE-Cy7, CD19 Pacific Blue (all Biolegend), NK1.1 PE-Cy7, CD3 FITC (eBioscience).

For intracellular detection of cytokines, the cells were stimulated with 100 ng/ml αGalCer (Biovision) for the indicated time and stained using Intracellular Fixation/Permeabilization Buffer Set and monensin (eBioscience) according to the instructions of the manufacturer. The following antibodies were used:

mouse: anti-Mouse IFNγ PerCP-Cy5.5, anti-Mouse IL-4 PE, anti-Mouse TNFα APC (all eBioscience).

human: anti-human IFNγ FITC, anti-human IL-4 PE, anti-human TNFα APC (all eBioscience).

Samples were measured on a Gallios flowcytometer (Beckman Coulter) and analysed using Kaluza 1.2 software (Beckman Coulter).

### Statistics

All statistical analyses were performed using GraphPad Prism 5.02 Software or IBM SPSS Statistics 21 software. Overall survival curves were plotted by Kaplan-Meier and the survival distributions of the different cohorts were compared using log-rank (Mantel-Cox) test. Mann-Whitney or Student's *t*-tests were used as indicated to analyse significance.

## SUPPLEMENTARY MATERIALS FIGURES AND TABLES


