# Supplementary material for: CD1d expression on chronic lymphocytic leukemia B cells affects disease progression and induces T cell skewing in CD8 positive and CD4CD8 double negative T cells
Source: Oncotarget. 2016 Jul 1;7(31):49459–69. doi: 10.18632/oncotarget.10372 (PMC5226521; doi:10.18632/oncotarget.10372)
Supplement: Supplementary file 1 [file oncotarget-07-49459-s001.pdf]

CD1d expression on chronic lymphocytic leukemia B cells affects disease progression and induces T cell skewing in CD8 positive and CD4CD8 double negative T cells

Supplementary Materials

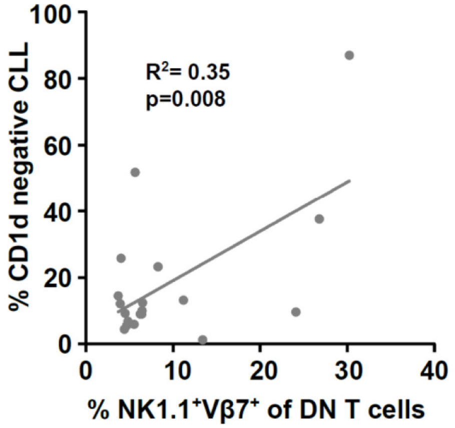

Supplementary Figure S1: Correlation of percentage of CD1d negative CLL cells with occurrence of NK1.1$^+$Vb7$^+$ cells within DN T cells in TCL1 mice.

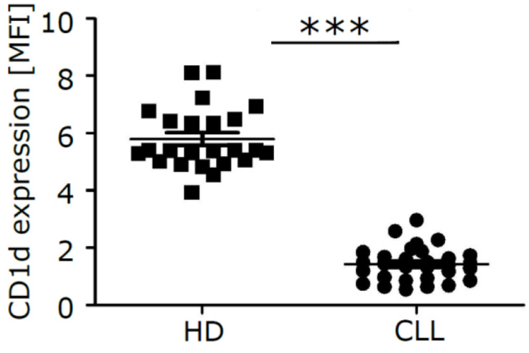

Supplementary Figure S2: CD1d expression on CLL cells. MFI of CD1d on human CLL samples shown in Figure 4C, 4D were calculated.

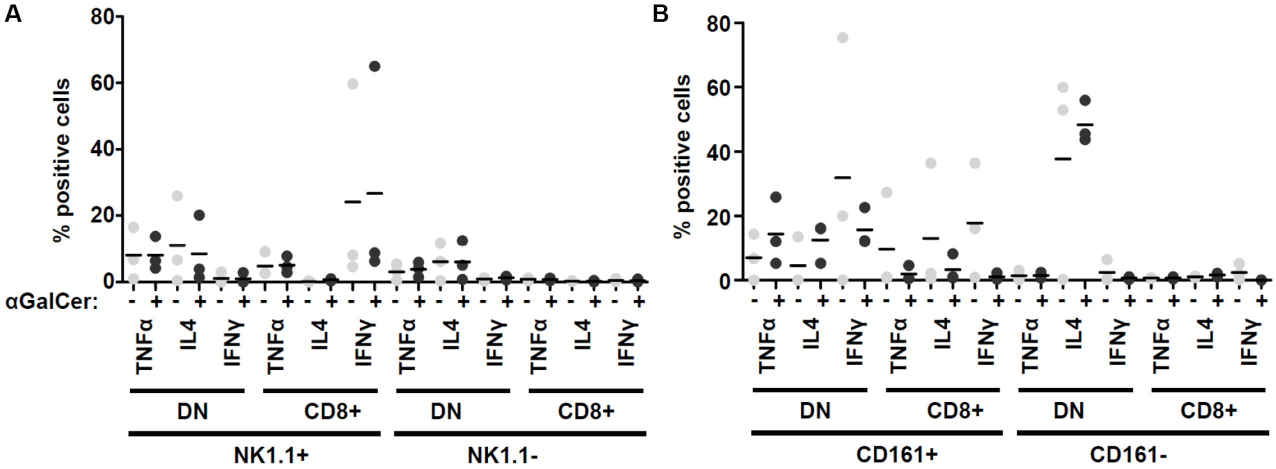

Supplementary Figure S3: Intracellular cytokine production of CD161 positive and CD161 negative CD8^{+} and DN CD3^{+} T cells upon αGalCer treatment *in vitro*. (**A**) Spleen cells from sacrificed TCL1 mice were incubated with/without αGalCer as indicated and the respective cytokines were determined 24 h post incubation. (**B**) PBMCs from CLL patients were stained for the presence of the respective cytokines 72 h post treatment with/without αGalCer.

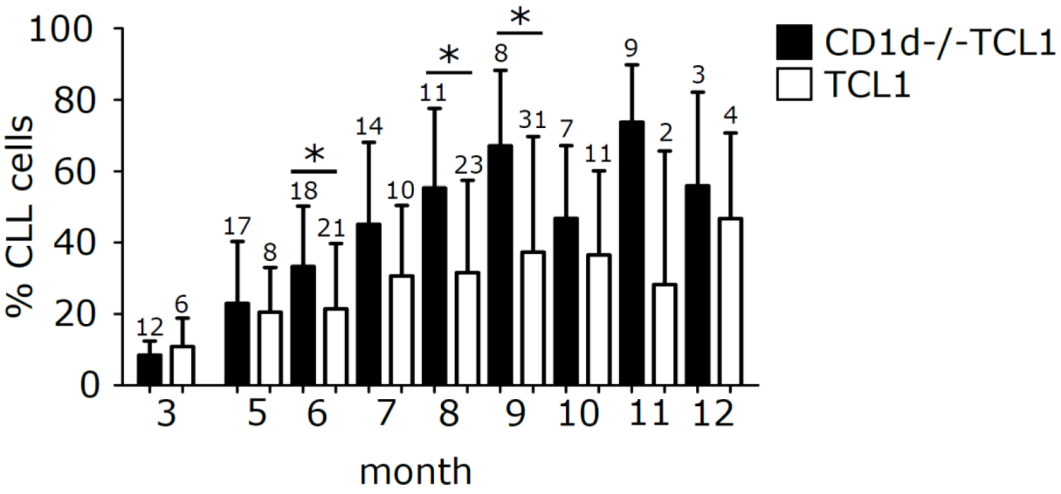

Supplementary Figure S4: Leukemic cell counts in tail vein blood from TCL1 and CD1d$^{-/-}$TCL1 mice. Mice were bled at the indicated age and leukemic cells were determined in tail vein blood (% CLL cells of lymphocytes). Significant differences are indicated with an asterisk ($p = 0.02$, Mann-Whitney *t*-test).

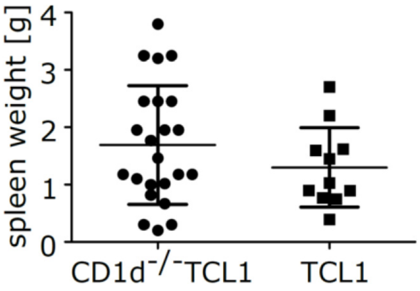

Supplementary Figure S5: Spleen weights of TCL1 and CD1d$^{-/-}$TCL1 mice.

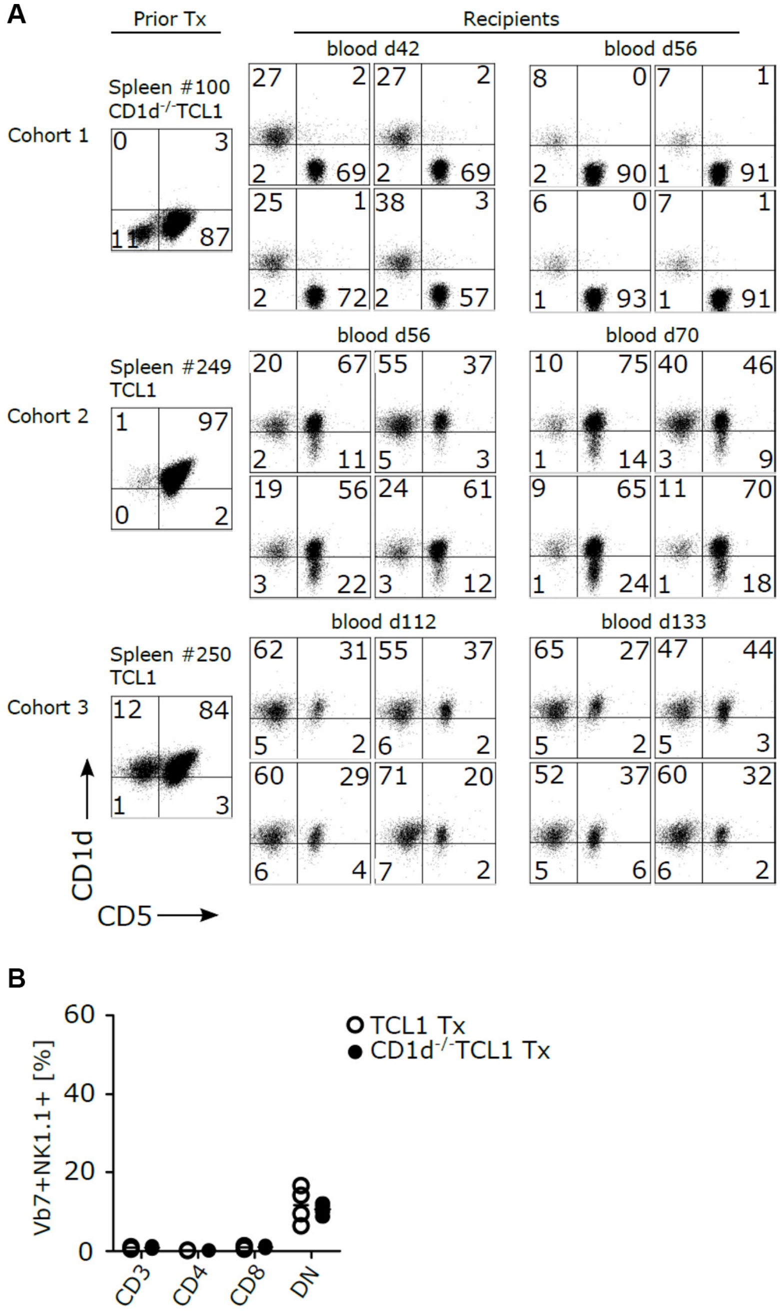

Supplementary Figure S6: CD1d expression and TCR-Vβ7 skewing upon tumor transplantation. (**A**) CD1d expression is shown for CD19CD5 positive tumors prior transplantation and for blood samples taken from the four recipients at the indicated time points post transplantation. Numbers within the squares of each FACS plot (gated on CD19^{+} lymphocytes) indicate percentages of the respective cell populations. Each FACS plot shows an individual mouse of the respective cohort. (**B**) Graph showing percentage of the respective spleen CD3^{+} T cell subset, which is expressing TCR-Vβ7 element at humane endpoints (TCL1 *n* = 4; CD1d$^{-/-}$TCL1 *n* = 4).

Supplementary Table S1: TCRVb CD161 staining of CLL samples shown in Figure 3

CLL#	Sample ID	Patient ID	Rai stage	ZAP 70	CD38	VLA-4 CD49d	Sex	Therapy	Time to diagnosis (ys)	Age at sampling (ys)	Expanded T clone	Expanded CD161+ clone	CMV Serum IgG
1	9935	777	I	high	high	high	M	chemonaive	1	61	yes	yes	+
2	9937	122	0	low	low	low	F	chemonaive	12	85	yes	yes	nd
3	9949	565	0	high	high	low	M	treated	4	65	yes	yes	+
4	9958	75	II	low	low	low	M	treated	25	62	yes	yes	−
5	9959	665	0	low	high	high	F	treated	6	78	yes	yes	+
6	9965	782	0	low	low	low	F	chemonaive	6	57	yes	yes	+
7	9967	771	0	nd	nd	low	F	chemonaive	0.3	73	yes	yes	+
8	9934	557	I	high	high	high	M	treated	4	61	yes	no	−
9	9951	578	0	low	low	low	F	treated	5	64	yes	no	−
10	9953	485	0	low	low	low	M	chemonaive	13	73	no	no	nd
11	9960	613	I	high	low	low	M	chemonaive	5	73	no	no	nd
12	9968	34	IV	nd	high	nd	F	treated	11	80	no	no	+
13	9932	668	0	high	low	low	M	chemonaive	3	40	no	no	+
14	9936	629	I	high	high	high	M	treated	3	73	no	no	−
15	9941	534	II	high	low	low	M	treated	5	44	no	no	−
16	9942	778	I	high	high	high	F	chemonaive	1	76	no	no	−
17	9950	780	I	high	low	high	M	treated	3	68	no	no	+
18	9952	700	I	high	low	low	M	chemonaive	2	49	no	no	−

nd: not determined.
ys: years.

Supplementary Table S2: Patient data for patients shown in Figure 4

Sample ID	Patient ID	Leukocyte count	% Lymphocytes	Age at sampling (ys)	Rai stage	ZAP 70	CD38	VLA-4 CD49d	Sex	Therapy	CMV serum IgG	CD1d negative CLL cells [%]
7779	283	10.15	61	65	II	high	low	low	M	treated	–	77,1
7780	442	22.86	84	65	0	low	high	low	M	chemonaive	nd	40,6
7783	109	65.9	86	86	0–I	low	low	high	F	chemonaive	+	8,2
7784	123	89.98	100	73	II	low	low	low	M	chemonaive	–	74,9
7786	204	101.14	92	72	0–I	low	high	high	M	chemonaive	–	43,4
7799	496	103.9	97	72	0–I	low	low	low	M	chemonaive	–	82,1
7800	230	109.81	87	76	0	high	high	high	F	chemonaive	+	51,8
7802	531	96.52	94	74	I	high	low	low	M	chemonaive	–	71,2
7805	337	74.06	80	70	I	low	high	low	M	treated	+	36,2
7809	400	23.6	73	77	0	low	low	low	F	chemonaive	+	72,2
7811	109	72.78	97	86	0–I	low	low	high	F	chemonaive	+	7
7814	573	16.45	68	59	nd	low	low	n. A.	F	chemonaive	–	65,6
7818	262	40.87	91	68	I	low	low	low	M	chemonaive	–	82,3
7819	223	11.89	57	70	0	low	low	low	M	chemonaive	nd	64,3
7820	92	26.87	79	59	0	low	low	low	M	chemonaive	+	84,7
7822	13	30.74	95	85	0–I	low	low	low	M	chemonaive	+	80,7
7823	435	210.5	98	64	II	low	low	low	F	chemonaive	–	93,8
7826	13	25.84	83	85	0–I	low	low	low	M	chemonaive	+	55,5
7832	175	22.16	49	73	0	high	low	low	M	chemonaive	–	28,7
7833	154	8.28	65	78	I–II	low	low	low	M	treated	–	54,7
7839	86	11.33	55	52	0	low	low	low	M	chemonaive	+	23,9
7845	230	103.17	94	76	0	high	high	high	F	chemonaive	+	85,3
7847	161	21.26	62	65	I	high	low	low	M	chemonaive	+	84,5
7848	549	19.92	74	79	I–II	borderline	low	low	F	chemonaive	–	63,3
7849	530	85.23	94	83	II	high	high	low	M	treated	+	88,7
7850	574	43.79	78	70	0	borderline	low	high	M	chemonaive	nd	40,7
7853	151	69.2	85	81	I	low	low	low	M	chemonaive	+	89,9
7856	575	33.2	78	79	II	low	low	low	M	chemonaive	nd	94,4
7858	93	12.5	61	79	0	low	low	low	F	chemonaive	+	77,9
7859	153	10.05	53	87	I	high	high	high	M	treated	borderline	73,4

nd: not determined.
ys: years.
Leukocyte count: ×1000/µl blood.
% Lymphocytes: % lymphocytes of leukocytes.

Supplementary Table S3: The CD1d neg

risk factor	*N* (risk group)	*P*-Value
leukocyte count	30	0.312
lymphocyte percentage	30	0.230
age	30	0.707
ZAP-70	17 (low)<br>11 (high)	0.069
CD38	23 (low)<br>7 (high)	0.693
VLA4 CD49d	22 (low)<br>7 (high)	0.020 *
Sex	21 (male)<br>9 (female)	0.489
Treatment status	25 (untreated)<br>5 (treated)	0.788
CMV serum IgG	11 (neg)<br>14 (pos)	0.537
RAI stage	10 (Rai 0)<br>6 (Rai 0–I)<br>6 (Rai I)<br>2 (Rai I–II)<br>5 (Rai II)	0.029 *

Data set was tested for normal distribution by the Kolmogorov-Smirnov normality test and the D'Agostino & Pearson omnibus normality test. Correlation analysis to leukocyte count, lymphocyte percentage and age were performed using Pearson correlation. Relation of CD1d negative CLL cells to ZAP-70, CD38, VLA4 subunit CD49d, Sex and CMV serum IgG groups were analysed by an unpaired *T*-test. Association to RAI stages was determined by ANNOVA analysis. *P*-Values < 0.05 were considered as significant.

Supplementary Table S4: NKT cell staining of CD1+/+(wildtype), TCL1, CD1d-/- and CD1d-/-TCL1 mice (from Figure 2D and 5D)

A

	CD3+	CD3+CD4+	CD3+CD8+	CD3+DN
CD1d+/+ (*n* = 6)	0.5% ± 0.2%	0.2% ± 0.2%	0.5% ± 0.2%	3.5% ± 3.1%
CD1d–/–(*n* = 4)	0.3% ± 0.2%	0.0% ± 0.0%	0.4% ± 0.2%	4.8% ± 1.5%
CD1d+/+TCL1(*n* = 6)	4.8% ± 3.4%	0.9% ± 1.0%	6.6% ± 5.3%	29.0% ± 14.8%
CD1d–/–TCL1 *n* = 5)	1.2% ± 0.7%	0.1% ± 0.2%	2.5% ± 1.8%	10.8% ± 5.8%

Percentage of NKT cell (CD3+, NK1.1+, TCR Vb7+) subsets (CD4+, CD8+ or DN) within different mouse cohorts. (mean % ± standard deviation).

B

	CD3+	CD3+CD4+	CD3+CD8+	CD3+DN
CD1d+/+TCL1 vs CD1d+/+	*p* = 0.005	*p* = 0.084	*p* = 0.005	*p* = 0.002
CD1d+/+TCL1 vs CD1d–/–	*p* = 0.01	*p* = nd	*p* = 0.01	*p* = 0.01
CD1d–/–TCL1 vs CD1d+/+	*p* = 0.034	*p* = 0.27	*p* = 0.013	*p* = 0.03
CD1d–/–TCL1 vs CD1d–/–	*p* = 0.05	*p* = nd	*p* = 0.02	*p* = 0.11
CD1d–/–TCL1 vs CD1d+/+TCL1	*p* = 0.028	*p* = 0.064	*p* = 0.142	*p* = 0.017

p-values of data from (A) as determined by Mann-Whitney test.
(nd: not definable as all splenocytes from CD1d–/– mice have 0% NKT cells within CD3+CD4+ cells).
